# How Do People Maintain Consensual Non-Monogamy? An International Development and Validation of the Multiple Relationships Maintenance Scale

**DOI:** 10.1007/s10508-025-03334-9

**Published:** 2026-03-16

**Authors:** Justin K. Mogilski, Geoffrey F. Miller, Peter K. Jonason, Katarzyna Grunt-Mejer, Jaroslava Varella Valentova, Rhonda Balzarini, David L. Rodrigues, Elisabeth Sheff, Laith Al-Shawaf, Zsófia Csajbók, Andrew G. Thomas, Tamas David-Barrett, Cezar Giosan, Daniel J. Kruger, David J. Ley, Justin J. Lehmiller, Heath A. Schechinger, Amy C. Moors, Stefano Ciaffoni, Leif Edward Ottesen Kennair, Stephen Whyte, Zuzana Štěrbová, Klára Bártová, Ryan G. Witherspoon, Magdalena Żemojtel-Piotrowska, Pavol Prokop, Virgil Zeigler-Hill, David P. Schmitt, Adil S. Sarıbay, Magdalena Lipnicka, Ivana Goláňová, Ezra Hampikian, William Costello, Limor Gottlieb, Cory J. Cascalheira, Michelle A. Larva

**Affiliations:** 1 Department of Psychology, University of South Carolina Salkehatchie, Walterboro, SC 29483 USA; 2 Psychology Department, University of New Mexico, Albuquerque, NM USA; 3 Psychological Research Institute, Vizja University, Warsaw, Poland; 4 Faculty of Psychology and Law in Poznań, University SWPS, Poznań, Poland; 5 Department of Experimental Psychology, University of São Paulo, São Paulo, Brazil; 6 Department of Psychology, Texas State University, San Marcos, TX USA; 7 Centre for Psychology Research and Social Intervention, Iscte-Instituto Universitário de Lisboa, Lisbon, Portugal; 8 Department of Psychology, University of Colorado, Colorado Springs, CO USA; 9 Portland, OR USA; 10 Pasadena, CA USA; 11 Department of Psychology and Life Sciences, Charles University, Prague, Czechia; 12 School of Psychology, Swansea University, Swansea, UK; 13 Trinity College, University of Oxford, Oxford, UK; 14 Department of Psychology, University of Bucharest, Bucharest, Romania; 15 Jacobs School of Medicine and Biomedical Sciences, University at Buffalo, Buffalo, NY USA; 16 School of Medicine, University of New Mexico, Albuquerque, NM USA; 17 The Kinsey Institute, Indiana University, Bloomington, Bloomington, IN USA; 18 University Health Services, University of California, Berkeley, Berkeley, CA USA; 19 Department of Psychology, Chapman University, Orange, CA USA; 20 Department of Psychology, University of Bologna, Bologna, Italy; 21 Department of Psychology, Norwegian University of Science and Technology, Trondheim, Norway; 22 School of Economics and Finance, Queensland University of Technology, Brisbane, Australia; 23 Department of Psychology, Faculty of Arts, Charles University, Prague, Czechia; 24 Institute of Psychology, Cardinal Stefan Wyszynski University in Warsaw, Warsaw, Poland; 25 Faculty of Natural Sciences, Institute of Zoology, Comenius University, Slovak Academy of Sciences, Bratislava, Slovakia; 26 Department of Psychology, Oakland University, Rochester, MI USA; 27 Department of Psychology, Kansas State University, Manhattan, KS USA; 28 Department of Psychology, Kadir Has University, Istanbul, Turkey; 29 Department of Psychology, John Paul II Catholic University of Lublin, Lublin, Poland; 30 Department of Psychology, Cambridge University, Cambridge, UK; 31 Department of Psychology, University of Texas at Austin, Austin, TX USA; 32 Department of Psychology, Brunel University London, London, UK; 33 Department of Counseling and Educational Psychology, New Mexico State University, Las Cruces, NM USA; 34 INVEST Flagship Research Center/Psychology, University of Turku, Turku, Finland

**Keywords:** Multiple relationships, Relationship maintenance, Consensual non-monogamy, Relationship quality, Relationship satisfaction

## Abstract

Maintaining multiple intimate relationships entails some risks and complications beyond exclusive pair bonding (i.e., monogamy). Consensual non-monogamy (CNM; e.g., polyamory, swinging, or open relationships) allegedly reduces these risks and complications, but no one has tried to comprehensively document typical CNM risk management practices, nor analyzed the relationship outcomes of those who use these practices. We developed the Multiple Relationships Maintenance Scale (MRMS) to examine how engagement in common CNM practices predicts relationship quality among people with one or more partners. In Study 1, a community sample of people with CNM experience (*N* = 429) nominated the best practices for maintaining CNM. Responses were combined with theory-derived practices to create the MRMS. In Study 2, we administered the MRMS in three international online surveys (*N* = 4,290) to assess factor structure and construct validity. Factor analysis revealed nine practices: disclosure of one’s extra-pair attractions to a partner, open communication about jealousy, compersion, willingness to care for unrelated children, shared extra-pair sexual experiences, partner (non)hierarchy, sexual health maintenance, thoughtful resource distribution among partners, and reputation management. Data from one of the three surveys (*n* = 871) showed that each practice had distinctive patterns of association with previous CNM experience, relationship and sexual satisfaction, partner investment and interdependence, and self-reported infidelity (i.e., how often extra-pair interactions feel to violate an existing relationship’s agreements). We discuss how the MRMS may be used to study how people in CNM relationships maintain multiple intimate relationships.

## Introduction

### Multi-Partnering and Relationship Quality

Multi-partnering is when an individual is concurrently in a romantic, sexual, or otherwise intimate relationship with more than one person. Contemporary humans mostly form monogamous pair bonds that are expected to be sexually exclusive (Schacht & Kramer, 2019). However, some people form and maintain several concurrent relationships (see Mogilski, 2022), such as in consensual non-monogamy (CNM; e.g., polyamory, swinging, and open relationships; Balzarini & Muise, 2020; Mogilski et al., 2023; Moors et al., 2021), plural marriage (e.g., Al-Krenawi, 2020; Li & He, 2021), casual dating (Garcia et al., 2018), sex work (Matolcsi et al., 2021; McCracken & Brooks-Gordon, 2021), or non-consensual practices such as infidelity (Haseli et al., 2019; Selterman et al., 2021).

Having multiple relationships is presumed to worsen the quality of relationships and family compared to monogamy. In their meta-analysis, Shaiful Bahari et al. (2021) showed that women in polygynous marriages (i.e., a man married to more than one woman) have higher rates of depression and anxiety, and children in polygamous households have higher rates of mental health problems and difficulty in school than those in monogamous marriages. Cultures with more polygyny also have higher rates of male-vs-male aggression and homicide, which may be caused by more violent competitiveness among men (Kruger et al., 2010), and higher child mortality (Ekholuenetale et al., 2020; Lawson & Gibson, 2018). Infidelity in socially monogamous pair bonds is linked to personal and social risks (Haseli et al., 2019; Selterman et al., 2023), including violence and homicide (Arnocky et al., 2022). Men whose partners engage in real or suspected sexual infidelity are also more likely to commit intimate partner violence (Goetz & Shackelford, 2009; Pinchon et al., 2020), and spouses report more abuse in households where husbands have affairs (Stieglitz et al., 2012). People who report a more unrestricted sociosexuality also report higher self-perceived risk of sexually transmitted infections (STIs) (Hall & Witherspoon, 2011) and fewer safer sex practices (Silva Júnior et al., 2022), and people with denser sexual networks are more likely to test positive for STIs (Helleringer & Kohler, 2007; Kenyon et al., 2018). Multi-partnering may thereby destabilize larger social networks because monogamy presumably constrains violent and otherwise zero-sum intrasexual competition by equalizing the distribution of available female partners (Brooks et al., 2022; Henrich et al., 2012), though this prediction has been criticized for overlooking the roles of female mate choice and female agency (Ross et al., 2018), socioecological factors (e.g., environmental vulnerability, access to education; Lawson et al., 2015), and other nuanced patterns of competition among the “surplus” unmarried men (Costello et al., 2024; Schacht et al., 2014).

Multi-partnering may thereby complicate intimate relationship maintenance. Having more than one intimate relationship demands more effort, time, energy, and/or money than only one relationship because cooperation beyond a monogamous dyad introduces more game-theoretic complications (Connor, 2010) and mating risks (Thomas et al., 2025), that impose more cognitive and communicative demands on those attempting to sustain mutually beneficial social exchange. The cognitive and emotional demands of romantic and sexual jealousy (Carson & Cupach, 2000; Elphinston et al., 2013), intimate attachment anxiety (Afifi et al., 2021), obsessive passion (Carswell & Impett, 2021), and other unique features of multi-partnering may be prohibitively burdensome for many people, given the demands of school, work, parenting, and daily life.

### Does Multi-Partnering Cause Worse Relationship Quality?

Nevertheless, multi-partnering itself might not be directly responsible for the distress nor instability commonly attributed to it. For example, infidelity arises predictably in response to–but does not reliably precede–lower relationship quality (Stavrova et al., 2023; Vowels et al., 2022; Weiser et al., 2022), and it can motivate partners to improve or dissolve a bad relationship. Those who openly solicit affairs (e.g., male users of the Ashley Madison website) report sexual dissatisfaction, need for autonomy, and desire for partner variety as stronger motives for their affairs than anger, lack of love, or neglect toward a partner (Selterman et al., 2023), and factors such as attachment insecurity are better predictors of infidelity motivated by anger, lack of love, neglect, and low commitment (Selterman et al., 2019). This suggests that the difficulty of predicting whether an additional relationship will affect the quality of an existing relationship causes more distress and partner conflict than simply having multiple intimate partners. In other words, uncertainty about a partner’s commitment, their continued fulfillment of expectations, or the future of the pair-bond (see Afifi & Reichert, 1996; Berger & Bradac, 1982; Knobloch, 2008) may be a more direct cause of the distress and instability attributed to multi-partnering.

For example, Weiser et al. (2014) found that secretive behavior and emotional fallout were perceived as most central to peoples’ concept of infidelity. Lehmiller (2009) showed that secrecy about extra-pair intimacy is related to worse relationship quality, possibly by reducing interpersonal closeness and increasing nervousness and fear. Similarly, Hangen et al. (2020) used latent profile analysis to differentiate types of non-monogamy based on levels of mutual consent, comfort, and communication among partners about other relationships. Monogamous and non-monogamous relationships with high levels of these three relationship dimensions reported higher relationship quality than non-monogamous relationships with mixed or low levels. This suggests that the dishonesty of infidelity (see Hughes, 2022) may cause more harm than having multiple partners.

Accordingly, open, coordinated, and comfortable exchange of information among partners may reduce distress caused by multi-partnering if this allows each partner to estimate how other relationships will impact an existing relationship’s quality. That is, if partners formalize a relationship agreement (Vilkin & Davila, 2023), identify the key concerns that extra-pair relationships introduce, and develop practices for managing these relationship threats, partners may prevent some of the most common conflicts that arise among those who maintain multiple partners.

### Does Consensual Non-Monogamy Resolve the Challenges of Multi-Partnering?

CNM provides a model for how people might design relationship agreements that accommodate multiple partners in stable, mutually satisfying ways. Many people in CNM relationships report stable, satisfactory relationships with the informed consent of all their partner(s) (Anderson et al., 2025). Like people in monogamous relationships, those in CNM relationships are demographically diverse (Balzarini et al., 2019a, 2019b, 2019c; Haupert et al., 2017; Levine et al., 2018), pursue emotional and sexual intimacy, commitment, passionate love, and shared goals with partners (Balzarini et al., 2019a, 2019b, 2019c; Rodrigues et al., 2017), have sex for both sexual and relational fulfillment (Mitchell et al., 2020; Wood et al., 2018), and build families (Palotta-Chiarolli et al., 2020). They also attempt to retain valued relationships (Mogilski et al., 2017, 2019), and troubleshoot relationship conflict (Brooks et al., 2022).

To address relationship conflict caused by multi-partnering, people in CNM relationships commonly communicate their relationship experiences and discuss how interactions with other partners occur. Prior research has identified some alleged ‘best practices’ and typical strategies for resolving conflict (Conley & Piemonte, 2020; Flicker et al., 2021; Klesse, 2019; Wosick-Correa, 2010). However, no one has comprehensively document these practices, created a measure to document individual differences in partners’ engagement in each practice, nor tested how people’s engagement in each practice is related to their relationship quality. Here we develop and begin to validate such a measure to characterize how the relationship maintenance practices of CNM address challenges introduced by multi-partnering.

Below, we outline five theoretically-derived challenges that emerge from multi-partnering: (1) partner loss and resource diversion, (2) pregnancy and childcare, (3) pathogen transmission, (4) zero-sum rivalry among partners, and (5) status loss. We also provide evidence for how people in CNM relationships attempt to address each challenge. This literature review was used to generate our measure’s hypothesized factor structure.

#### Partner Loss and Resource Diversion

Extra-pair bonds can threaten established patterns of resource sharing among partners (e.g., sharing money, chores, and childcare). Connecting with others is a fundamental human motive (Baumeister & Leary, 2017) as it facilitates the formation of dependable social bonds for sharing resources that would be difficult to acquire or defend alone (e.g., buying a house, running a business, raising children). This interdependence allows partners to align their behavior more easily toward shared goals (Rusbult & Buunk, 1993). When such established sharing patterns (e.g., in marriage or cohabitation) are disrupted or become unfair or inadequate because of a perceived threat, partners will be motivated to defend a valued relationship against that threat.

Indeed, the emotion of jealousy functions to defend against significant threats to a valued relationship (Chung & Harris, 2018), and upregulates mate retention (Davis et al., 2018). Partners who experience jealousy, particularly in response to an actual–rather than suspected–relationship threat, report higher relationship satisfaction, commitment, and attachment security (Attridge, 2013; Barelds & Barelds-Dijkstra, 2007; Buunk & Dijkstra, 2021), because jealousy encourages partners to troubleshoot relationship problems. However, jealousy can be harmful to relationship quality when it is chronic (Rydell & Bringle, 2007), poorly regulated (De Cristofaro et al., 2022; Kyegombe et al., 2022), or triggers conflict or abuse (e.g., Toplu-Demirtaş et al., 2022).

In CNM, people report diverse practices for managing the threat of partner loss, including open communication about jealousy (Buunk, 1982; Philpot et al., 2018), reframing of negative emotions (de Visser & McDonald, 2007; e.g., cognitive reappraisal, Blake et al., 2018), fostering compersion (i.e., enjoyment of a partner’s extra-pair relationships; Balzarini et al., 2021; Flicker et al., 2021; Hunter & Stockwell, 2022; Mogilski et al., 2019), or planning how limited resources (e.g., time, attention, money) will be shared among partners (Andersson, 2022; Wosick-Correa, 2010). These practices presumably address the threat of resource diversion by reducing uncertainty about attachment and investment. That is, if partners are mutually aware of and responsive to each other’s concerns about potential diversion of valuable resources, partners can work to address these concerns.

Because a partner’s other relationships can create uncertainty about how time, money, and effort will be allocated to each partner, people in CNM relationships sometimes designate specific partners as “primary” and others as “secondary” or “tertiary.” This is termed relationship hierarchy and is typical of most CNM relationships (Balzarini et al., 2017, 2019a, 2019b, 2019c; Mogilski et al., 2017, 2019, 2020; Moors et al., 2019), though it may be less common in certain types of CNM (e.g., polyamory or “relationship anarchy,” which explicitly rejects hierarchy; Conley & Piemonte, 2020; Flicker et al., 2021). People may establish relationship hierarchy because it defends the advantages of privileging a partner. That is, if losing a partner’s investment would be especially costly (e.g., due to marriage, mortgage, children, or a family business), then giving them preferential access to one’s time, attention, material wealth, and other shared resources–and restricting others’ access to these same resources–may prevent a partner’s disinvestment and reduce jealousy.

#### Pregnancy and Childcare

From an evolutionary perspective, extra-pair relationships can introduce serious uncertainty about shared parental investment in raising children. In the biological sciences, ‘cuckoldry’ occurs when an individual unwittingly invests in unrelated offspring. This may disrupt reproductive success if such parenting effort could instead be invested into related offspring or other avenues tributary to reproductive success. Indeed, mate guarding (or sexual proprietariness; Wilson & Daly, 1996) may have evolved to address cuckoldry and mate poaching more generally (Buss & Shackelford, 1997), and some have argued that minimizing cuckoldry underlies monogamy (Lukas & Clutton-Brock, 2013; Schacht & Bell, 2016). Therefore, people may avoid multiple intimate relationships to prevent unrelated partners from interfering with parental investment and childcare.

Childcare among those with multiple intimate relationships is empirically understudied. Most research compares child wellbeing in polygynous versus monogamous families in sub-Saharan Africa and the Middle East. This work suggests that polygyny, on average, produces worse childhood outcomes (Al-krenawi & Slonim-Nevo, 2008; Bove et al., 2012; Eldebour et al., 2003). However, such studies often do not account for parental education, socioeconomic status, and other family- and community-level characteristics that better explain the worse childhood outcomes in polygynous families (Al-Sharifi et al., 2016; Elbedour et al., 2002; Lawson & Uggla, 2014). The role of such confounds is supported in some samples (Lawson & Gibson, 2018; Omariba & Boyle, 2007) but not others (Adedini & Odimegwu, 2017; Amare et al., 2021), suggesting that multi-partnered parenting entails additional challenges for childcare that may be offset or amplified by family, relationship, and community norms (also see Shaiful Bahari et al., 2021).

People in CNM–and particularly polyamorous–relationships may adopt relationship norms that promote positive childcare outcomes. Compared to research on polygyny, there is little quantitative research assessing how polyamorous family practices influence childhood outcomes, but published interviews suggest that polyamorous parenting is commonly successful and can offer children broader access to material and social capital (Pallotta-Chiarolli, 2006; Sheff, 2014) when extra partners function as ‘alloparents’. Nevertheless, CNM partners typically form parenting agreements to reduce conflict over resources and childcare duties. Partners may discuss expectations for shared parenting, negotiate how caregiving responsibility will be allocated, or plan how resources will be provisioned among partners, extended families, and children (Pain, 2020). In this way, polyamorous families and close-knit networks (e.g., “polycules”) may serve the conventional role of providing stable and secure care for children via alternative family practices (Pain, 2020; Pallotta-Chiarolli et al., 2020; Roodsaz, 2021; Schadler, 2021).

#### Pathogen Transmission

Communicable pathogens are a threat to the health and safety of individuals and groups. People who have multiple, concurrent sexual partners report a higher rate of STIs than those with a single partner (Joffe et al., 1992), and features such as the interconnectedness of one’s network of sexual partners can increase STI spread (Amirkhanian, 2014; Doherty et al., 2005; Ward, 2007). Monogamy may protect against the spread of STIs if partners faithfully avoid extra-pair sex (Conley & Piemonte, 2020), but research suggests that monogamy agreements are less effective against the spread of STIs than presumed (Swan & Thompson, 2016). For example, though strict monogamy constrains the sexual network across which STIs may transmit, imperfect monogamy (e.g., infidelity) appears to be riskier than planned non-monogamy with respect to STI transmission. People committing infidelity are less likely to practice safe sex than people who multi-partner with the informed consent of their partner(s) (Conley et al., 2013; Lehmiller, 2015). However, it also appears that some types of CNM impose greater risk than others (Platteau et al., 2017; Rodrigues et al., 2019a, 2019b), because different CNM relationship types and subcultures can have different sexual health customs (Levine et al., 2018). Factors such as sexual network connectivity (Doherty et al., 2005; Kenyon, 2017), sexual mixing patterns (Aral et al., 1999; Garnett & Anderson, 1996), sexual motivations (Rodrigues et al., 2022), and failure to use precautionary sexual health tools (e.g., STI testing, condoms; Steiner et al., 2021) are stronger predictors of disease transmission than multi-partnering per se. This suggests that how partners communicate about sexual risk, and whether they use “best practices” in sexual health maintenance, could buffer against the increased STI risk of having multiple intimate partners.

#### Zero-Sum Rivalry

Competition for reproductive partners is ubiquitous in sexually reproducing organisms (Krems et al., 2023) and may be more intense within promiscuous mating systems unconstrained by monogamy (e.g., Davis et al., 2023). This competition can be zero-sum, such that one individual succeeds at a cost to another. However, romantic partners may also find cooperative, positive-sum solutions for resolving conflict while multi-partnering. In their biographical review of well-known Western intellectuals and artists, Watson and Lubrano (2021) document collaborative behaviors between “metamours” (i.e., people who are each intimately involved with the same individual) and argue that these offer an alternative to sexually competitive behavior. Conley and Piemonte (2020) likewise found that people in CNM relationships who report greater familiarity with their metamours (i.e., their partner’s partners) reported greater relationship satisfaction and intra-pair partner trust.

Some clinicians and therapists working with plural marriages attempt to foster collaboration among metamours to mitigate the costs of competitiveness on relationship quality and social network wellbeing. For example, Al-Krenawi (1998) found that polygamous co-wives who were counselled to communicate with one another and establish friendships reported improved relationship satisfaction and family functioning. Similarly, in polyamorous relationships, partners and metamours may collaboratively address negative emotions caused by perceived inequity (e.g., jealousy, anger, or envy; Kolmes & Witherspoon, 2017; Sprott et al., 2017), establish relationship hierarchy to manage insecurity (Flicker et al., 2021; Johnston, 2022), or negotiate how time, attention, and resources are divided among partners. These practices may allow partners to anticipate conflict, devise equitable solutions, and troubleshoot transgressions, quelling partner competitiveness and conflict.

#### Status Loss

Having multiple intimate partners can help or hurt one’s social status. Stigma against CNM is widespread (Moors & Ramos, 2022; Rodrigues, 2024), and people perceive those in CNM relationships as having more undesirable traits (e.g., poor intelligence, morality, and relationship skills) compared to those in monogamous relationships (Balzarini et al., 2018; Grunt-Mejer, 2016; Moors et al., 2021; Rodrigues et al., 2018, 2021). If those with multiple partners are presumed to be less cooperative, or to have more unstable relationships, people may rationally avoid affiliating with them to minimize risk. Conflict among partners is the most salient concern among those who report greater apprehension toward CNM (Cunningham et al., 2022). Likewise, people with more prejudice against CNM are more likely to consider a relationship’s status implications and practical features (i.e., Pragma love), such as family approval, similar partner background, or how a partner reflects on their career (Flicker & Sancier-Barbosa, 2022).

Kurzban and Leary (2001) argue that people are excluded from social interaction (i.e., stigmatized) when they are either deemed a poor social exchange partner, prevent access to in-group identity and its benefits, and/or are perceived as a vector for pathogens. In this view, stigmatizing others solidifies one’s commitment to the range of acceptable actions that protect in-group members from free-riders, out-group hostility, and disease. People may therefore justify stigma against sexual promiscuity when they perceive that there are insufficient safeguards to the social challenges of having multiple partners (e.g., effective birth control; sexual health management; thoughtful allocation of one’s time, attention, and physical investment among partners).

In response, people may be secretive about multi-partnering to reduce reputational risk (Piazza & Bering, 2010). People in CNM relationships often report hiding their relationship(s) from non-partners (e.g., Füllgrabe & Smith, 2023) – especially their secondary relationships (Balzarini et al., 2017, 2019a, 2019b, 2019c). However, such secrecy is associated with worse relationship quality (Balzarini et al., 2019a, 2019b, 2019c; Foster & Campbell, 2005; Lehmiller, 2009). By contrast, those who are open about their non-monogamy with others can potentially gain access to social support (e.g., therapy, healthcare) that buffers against negative outcomes (Schechinger et al., 2018; Vaughn et al., 2019). Knowing how people successfully form several, concurrent intimate relationships–what works and what doesn’t–could reduce the perceived need to stigmatize CNM and thereby reduce the distress and conflict that multi-partnering may cause.

#### Current Research Aims

Here, our research aims were to first (*RA1*) identify how people in CNM relationships manage the risks and complications noted above, such as jealousy and abandonment risk, resource and childcare disinvestment, STI transmission, rivalry among partners, and social stigma. Then, (*RA2*) we created a self-report inventory to measure how often people engage in these CNM relationship maintenance practices within their relationship(s). We then validate the measure by (*RA3*) factor analyzing the measure’s items, and (*RA4*) assessing whether people with more CNM experience report more engagement in these practices than those without experience. We also (*RA5*) test if scores on this measure are related to validated measures of relationship quality with a partner, including intimacy, passion, and commitment. Finally, to assess whether CNM is distinct from non-consensual forms of multi-partnering (i.e., infidelity), we examine (*RA6*) whether people who engage in these practices are less likely to feel that their interactions with other people are infidelity, and (*RA7*) whether self-reported infidelity is associated with relationship quality. A list of project hypotheses was preregistered with the Open Science Framework (https://osf.io/wex2f); however, only hypotheses relevant to the research aims of this paper were tested, and several unplanned hypotheses were performed post hoc.

We first created an expert list of CNM “best practices” from the literature review and the expertise and personal experiences of an interdisciplinary team of evolutionary, relationship, and sexuality scientists and therapists (see Author Contributions below). These were our Study 1 hypothesized factors (see Table 1). To verify their validity, we recruited a community sample of people with CNM experience to nominate the best and worst practices for managing conflict among multiple partners. These nominations were synthesized with the hypothesized factors to create an expected factor structure of ten relationship maintenance practices (Table 2) (*RA1*). From this, we formalized the thirty item Multiple Relationships Maintenance Scale (MRMS) (*RA2*).

**Table 1:** Study 1 hypothesized relationship maintenance practices and descriptions generated prior to the start of data collection

1) Adherence to Relationship Agreement
Description: How closely do you follow the agreements that you and your partner have about relationship exclusivity?
2) Intra-pair Positive Inducements
Description: How often do you make effort to improve your partner(s) well-being? Improve yourself to become a more attractive mate? Produce costly or hard-to-fake signals of relationship investment and commitment?
3) Information Sharing
Description: How well do you share information with your partner that is relevant to managing multiple romantic partnerships?
4) Desire Receptivity and Fulfillment
Description: How do you respond to your partner’s attractions to other people? Do you encourage them to act on these attractions? Do you invest time to understand their perspective?
5) Sensitivity to Limits and Anxieties
Description: How do you talk with your partner about anxieties related to multi-partner mating? How do you help resolve your partner’s anxieties? Do you make an effort to resolve conflict between your partners?
Notes: Jealous anxiety, sexual orientation + gender identity concerns, anxiety that the partner is not attracted to you, bullying + intrasexual competition among partners. Jealousy management: avoidance, mindfulness, eroticization
6) Consent-seeking
Description: Do you explicitly seek your partner’s consent before flirting or becoming intimate with someone else? Do you allow your partner to revoke this consent?
Notes: Reversibility (i.e., consent can be withdrawn at any time). Willingness (or capacity) to regulate impulsive desires to maintain relationship and personal health. Matching behavior to what partner has consented to
7) Respect for and Preference of Hierarchy
Description: Do you rank your romantic partners (e.g., primary/secondary)? If a potential partner has a similar hierarchical agreement with a current partner, do you respect their agreement? Do you have a preference for hierarchical multi-partner relationships?
Notes: How rigid is the relationship structure? Do you respect that structure and its boundaries? Are you currently defecting on an established relationship’s social contract? Are you helping someone else defect? How often do you ensure a current partner that they are superior (i.e., primary) compared to another partner (i.e., secondary)
8) Sexual Health Maintenance
Description: How much effort is taken to avoid contracting or transmitting sexual disease?
9) Reputation Management
Description: Do you hide your relationships with multiple partners from other people? If your partner is concerned about what other people would think of your having multiple romantic partners, are you discreet? Do you respect a partner’s expectation for confidentiality/privacy? Do you respect your partner’s preference to be seen or known as your partner to others?
10) Childcare Management
Description: If you or your partner have children, do you take precaution to manage how children are affected by your relationships? Do you negotiate childcare responsibilities among you and your partners? Do you take precaution to avoid unintended pregnancy?
11) Shared Sexual Experience
Description: Do you include your partner(s) in sexual activity? How well do each partners’ sexual fantasies and kinks synergize (e.g., does a woman’s interest in extra-pair sex help fulfill her husband’s fantasy to be cuckolded?)

**Table 2:** Relationship maintenance practices and descriptions identified after Study 1 data collection and expected to emerge from factor analysis of Study 2 data

Practice	Description
Exclusivity agreement	Tendency to share and discuss expectations about intimate, extra-pair relationships with a partner. Those who score higher tend to communicate their exclusivity preferences with their partner(s). Those who score lower tend not to explicitly discuss exclusivity with their partner(s)
Disclosure of Extra-pair attraction	Tendency to tell a partner about extra-pair attractions. Those who score high will tend to frequently and accurately reveal their attractions to other people. Those who score low will tend to hide this information
Compersion	Tendency to enjoy when a partner acts on their extra-pair attractions (e.g., has sex or falls in love with another person). Those who score higher will enjoy or find pleasure in partner’s sexual or otherwise intimate involvement with other people. Those who score lower will tend not to enjoy their partner’s extra-pair interactions
Jealousy regulation	Tendency to feel that one can communicate with a partner about jealousy, pregnancy concern, fear of abandonment, etc. Those who score higher will feel freer to discuss relationship jealousy with their partner. Those who score lower will be more hesitant to do so
Partner hierarchy	Tendency to rank romantic partners. Those who score higher will tend to prefer relationships that are rigidly structured with some partners receiving priority over others. Those who score lower will tend to prefer relationships with greater egalitarianism among partners
Sexual health maintenance	Tendency to avoid sexual disease and unwanted pregnancy. Those who score higher will tend to take precaution against disease or regulate pregnancy. Those who score lower will tend to ignore the risk of infection and accidental pregnancy
Shared extra-pair sexuality	Tendency to include partner(s) in sexual activity with other people. Those who score higher will tend to involve their partner in sexual activity. Those who score lower will tend not to involve their partner(s) in sexual activity
Childcare management	Tendency to share childcare responsibilities with one’s partner(s)(if a parent). Those who score higher will tend to involve their partner(s) in childcare. Those who score lower will tend to limit partner(s) involvement
Reputation management	Tendency to hide or be publicly open about one’s relationship(s) with other partners and third-parties (e.g., friends and family). People who score higher will tend toward hiding their partners and interactions. People who score lower will tend toward public disclosure of their relationships
Resource distribution among partners	The tendency to consider how one’s resources (e.g., time, money, attention) are distributed among romantic or sexual partners. People who score higher will tend to plan how they spend personal resources on their partner(s) relative to other targets of investment (e.g., friends, family, other partners, community, work, personal leisure, etc.). Those who score lower will tend not to plan

In Study 2, the MRMS was then administered in three surveys. Each survey assessed a different aspect of relationship functioning: (1) relationship quality, (2) partner conflict, and (3) physical and mental health. Surveys were administered separately to reduce participant fatigue. Here, we combined responses to all three surveys to assess the factor structure of the MRMS (*RA3*) and to assess whether people with more experience with CNM engage more in the MRMS practices (*RA4*). Then, we used only the relationship quality survey to determine whether those who engage more in each practice experience more intimacy, more passion, and more commitment with their partner(s), particularly people with multiple intimate partners. Conversely, we expected that more self-reported infidelity would be related to less engagement in the MRMS practices (*RA6*) and less intimacy, less passion, and less commitment (*RA7*).

## Study 1

## Method

### Participants

Participants experienced with CNM were recruited from social media websites (e.g., Facebook, Reddit, Twitter/X) and researchers’ personal networks, and submitted all responses via Qualtrics online survey software. All survey materials were presented in English. Participation was not compensated financially, though some university students were issued partial course credit in exchange for participation. Participants were excluded if they did not nominate at least one practice for managing conflict in a CNM relationship. This resulted in a final sample of 429 (age: *M* = 37.19, *SD* = 10.97, range = 19–81). Participants self-identified their sex, gender, relationship structure, ethnicity, and world region of origin (see Table 3).

**Table 3:** Demographic information for Study 1 and Study 2 participants

	Study 1 (*N* = 429)	Study 2 (Full sample: *N* = 4,290)	Study 2 (Relationship quality survey only: *n* = 871)
*Survey Language*			
English	100%	39.5%	69.9%
Portuguese (Brazil)	–	14.6%	14.7%
Portuguese	–	0.3%	0.5%
Finnish	–	14.5%	3.3%
Italian	–	28.7%	5.5%
Polish	–	2.5%	6.1%
*Sex*			
Female	66.2%	65.5%	60.90%
Male	33.3%	34.3%	39.00%
Intersex	0.5%	0.2%	0.10%
*Gender*			
Men	31.5%	32.8%	36.5%
Women	56.2%	57.4%	52.8%
Non-binary	7.0%	3.7%	4.0%
Genderqueer	3.0%	1.5%	1.3%
Transgender	0.5%	0.8%	0.4%
Other	1.6%	5.4%	4.0%
*Relationship Identity*			
Polyamorous	54.3%	33.2%	39.4%
Open	20.0%	12.3%	16.0%
Single	11.0%	–	–
Swinging	9.3%	6.9%	11.3%
Monogamous	5.4%	47.5%	33.3%
*Current # of Partners*			
Single-partnered	–	41.6%	45.5%
Multi-partnered	–	58.4%	54.5%
*Sexual Orientation*			
Heterosexual	–	54.7%	46.5%
Bisexual	–	21.5%	27.9%
Pansexual	–	8.8%	10.5%
Homosexual	–	4.7%	5.1%
Other	–	10.2%	9.7%
*Ethnicity*			
White	86.7%	87.2%	80.8%
Hisp./Latin/Spanish	–	3.7%	5.7%
Asian	4.2%	1.9%	3.5%
Black	2.3%	3.7%	5.2%
Indigenous	0.5%	0.4%	1.1%
Other	5.6%	2.4%	3.6%
*World Region Origin*			
North America (United States, Canada)	61.0%	20.9%	40.1%
Europe (Albania, Andorra, Austria, Belgium, Croatia, Czechia, Denmark, Finland, France, Germany, Greece, Ireland, Hungary, Italy, Latvia, Netherlands, Norway, Poland, Portugal, Romania, Russia, Serbia, Slovakia, Slovenia, Spain, Sweden, Switzerland, Turkey, Ukraine, United Kingdom)	24.4%	59.9%	32.7%
Asia (Bangladesh, China, India, Japan, South Korea, Malaysia, Nepal, Pakistan, Singapore, Thailand, Kazakhstan)	7.5%	1.1%	2.7%
Middle East (Azerbaijan, Iran, Israel, Lebanon, United Arab Emirates)	2.6%	0.1%	–
South/Central America (Argentina, Bolivia, Brazil, Chile, Colombia, Ecuador, El Salvador, Guatemala, Mexico)	1.4%	16.7%	17.6%
Africa (Nigeria, South Africa)	–	0.4%	0.4%
Oceania (Australia, New Zealand)	0.9%	1.0%	2.1%

### Measures and Procedure

Prior to data collection, we developed an hypothesized factor structure (Table 1) for the MRMS based on (1) project collaborators’ personal knowledge of common CNM relationship challenges and their potential solutions, (2) direct correspondence with people in CNM relationships, and (3) the published research on CNM relationship maintenance reviewed in the introduction to this paper.

After informed consent, participants reported their demographic information, and then received three sets of open-ended questions. First, they were asked to describe three challenges that they or other people have experienced while pursuing multiple, concurrent romantic partners. Then, they were asked to think about these challenges and write down five of the most effective practices for resolving these challenges. Finally, they were asked to write down five of the least effective practices for resolving these challenges. The order in which participants were asked to report the most and least effective practices was counterbalanced.

## Results

Participants’ responses were sorted by four independent sorters. Each sorter saw the full list of participants’ most effective practices. Sorters were instructed to categorize each practice into one (or more) of the 11 hypothesized factors described in Table 1. A definition of each factor was provided. Practices that could not be categorized into one of the factors were designated “uncategorized”. The hypothesized factors were then revised to account for practices that did not fit the existing array of factors, and the independent sorters were asked to sort the remaining uncategorized practices into the new factor list. From this, we generated a final factor structure of 10 practices that we expected to emerge during the Study 2 factor analysis (see Table 2). Three items were drafted for each of these 10 expected factors to create two versions of the MRMS, each containing 30 items (see Appendix A). The MRMS-self asks participants how well each item generally describes them, whereas the MRMS-partner asks how well each item describes their relationship with a specific partner.

## Discussion

In Study 1, we identified 10 practices that people in CNM relationships use to manage the risks and complications of multi-partnering (*RA1*). From this, we drafted the MRMS (*RA2*). In Study 2, we addressed *RA3-7* by administering the MRMS and measures of relationship quality to a large international sample of people in monogamous and CNM relationships.

## Study 2

## Method

After drafting the MRMS, the authors discussed which other predictor and outcome variables should be included in the three validation surveys, including the demographic questionnaire and each set of outcome variables. Responses to the demographic questionnaire and each MRMS-self and MRMS-partner measure from all three surveys were pooled to perform factor analyses. Only responses to the relationship quality survey were used to assess how scores on the MRMS relate to relationship quality (i.e., intimacy, passion, and commitment).

### Participants

Each survey was administered via (1) promoted social media advertisement on Facebook and Twitter/X, (2) snowball sampling via project members’ personal networks (e.g., undergraduate research participant pools, email listservs, personal social media accounts), and (3) posts to social media websites frequented by people in CNM relationships (e.g., the r/polyamory and r/swingers sub-Reddits). Participants were not financially compensated. The surveys were completed in English, Portuguese (Brazil), Portuguese (Portugal), Italian, Polish, and Finnish. The original survey was written in English and was translated by 2–5 native-speaking professional translators, researchers, and/or non-researchers. These translations were then merged into a single translation and then back translated into English. Published translations for each outcome measure were used if available. Participation was not compensated financially, though some university students were issued partial course credit in exchange for participation.

Participants were asked in each survey to generate an anonymous, eight-unit identification code by entering letters and numbers from personal information (e.g., the first two letters of their name; the last two letters of their mother’s first name). This was used to identify and eliminate duplicate entries (*n* = 96), yielding 4,290 participants (age: *M* = 36.46, *SD* = 13.01, range = 19–83) who completed the full MRMS-self and MRMS-partner measure for at least one partner across all three surveys. We used a subset of this sample who had completed the MRMS and all relationship quality measures (*n* = 871; age: *M* = 33.06, *SD* = 12.58, range = 18–83) to assess how MRMS scores were related to relationship quality. No other procedures were used to screen participants from the analytic sample. Demographic information for each dataset is presented in Table 3.

### Measures and Procedure

All survey materials were administered using Qualtrics survey software. After giving informed consent, participants provided demographic information, reported their relationship identity (i.e., polyamorous, open, swinging, and monogamous relationships), their experience having been in each type of relationship (1 = *none*, 7 = *extensive*), and they completed the MRMS-self. Participants then provided the first name of up to four current partners and answered questions about the first two. Participants first reported their partner’s demographic information (i.e., age, sex, gender, sexual orientation, how long they had been romantically or sexually involved, and whether their relationship agreement permitted them or their partner to have sexual contact or form emotionally intimate attachments with other people). Then, they briefly described which actions they consider “infidelity” or “cheating” in their relationship with their partner. After, they reported how often they: (1) have interactions with other people that feel like infidelity, (2) keep their sexual interactions with other people a secret from their partner, and (3) keep their emotional attachments with other people a secret from their partner (1 = *never*, 5 = *always*). They were also asked how often they felt that their partner performed these actions. Participants then completed the MRMS-partner about their relationship with their partner.

In the relationship quality survey participants also completed the following relationship satisfaction measures about each partner: the perceived relationship quality components scale (PRQC; Fletcher et al., 2000; *α*_*p1*_ = .94, *α*_*p2*_ = .94), past and planned relationship investments (Goodfriend & Agnew, 2008; past: *α*_*p1*_ = .89, *α*_*p2*_ = 91; planned:; *α*_*p1*_ = .74, *α*_*p2*_ = .94), the new sexual satisfaction scale (NSSS; Štulhofer et al., 2010; *α*_*p1*_ = .94, *α*_*p2*_ = .94), measures of eroticism (*α*_*p1*_ = .94, *α*_*p2*_ = .96) and nurturance (*α*_*p1*_ = .91, *α*_*p2*_ = .91; Balzarini et al., 2019a, 2019b, 2019c), and interdependence measures (Knobloch & Solomon, 2004; interference: *α*_*p1*_ = .85, *α*_*p2*_ = .90; facilitation: *α*_*p1*_ = .88, *α*_*p2*_ = .92). Participants completed all questions about one partner before answering the same questions about the other partner. Which partner they rated first was randomized. The relationship satisfaction measures were also presented in random order.

## Results

### What is the MRMS Factor Structure? (*RA3*)

To address *RA3*, we used principal axis factoring with promax rotation in SPSS v.26 to explore the factor structure of the relationship quality survey participants’ responses to items of the MRMS (N = 871). Eight factors yielded eigenvalues > 1 and explained 61% of the variance. The scree plot also showed this number of factors was reasonable. The rotated eight-factor solution contained multiple items with high cross-loadings (i.e., the items had higher loading on the secondary factor than .5 times the loading on the primary factor; items: 1, 2, 9, 20, and 25). Also, Items 17 had low primary factor loading (below .30).

After eliminating these six items, we performed a confirmatory factor analysis (CFA) on the 24-item, eight-factor model using the full sample (*N* = 4,290). The model fit indices were accepted if RMSEA and SRMR were lower than 0.08, and CFI and TLI were higher than 0.90 (Brown, 2006). Measurement invariance was tested across self-reported sex (i.e., male or female), number of partners (single- or multi-partnered), relationship identity (i.e., monogamous or CNM), world region of residence (i.e., North American or European), language (English or non-English), and age (younger than 40 or 40 and over). Scalar measurement invariance was considered established if the increase in RMSEA was not higher than .015, and the decrease in CFI and TLI was not higher than .01, when compared between the configural versus metric models, and metric versus scalar models (Chen, 2007). Maximum Likelihood Robust estimator was used because some items had a skewed distribution.

The model fit indices did not support the eight-factor solution, *χ*^*2*^(225) = 1090.97, RMSEA = .06 (90% CI [.05-.06]), CFI = 0.90, TLI = 0.88, and SRMR = 0.05. This result was possibly observed because the first factor contained more items than any other factor. We performed a subsequent EFA on the subset of eight items (i.e., Items 1, 2, 3, 4, 5, 10, 11, and 12), which identified two separate factors. After the split, a nine-factor, 24-item model was submitted to CFA (see Table 4 for factor loadings). The residual variance of Item 7 was again fixed at zero to account for negative, non-significant residual variance. The yielded model fit was excellent (Table 5). The first two latent factors (the split products of the large original first factor) were modestly correlated, *r* = .47, *p* < .001, well below the threshold (*r* > .80–.85) where discriminant validity is typically considered compromised due to factor redundancy (Brown, 2006). Then, we investigated whether the nine-factor model was invariant across sex, number of partners, relationship identity, country of residence, survey language, and age. The residual variance of certain items needed to be relaxed within the small size subgroups (Table 5), but scalar measurement invariance was established across all grouping variables (Table 6). Standardized group means from each multigroup comparison’s scalar model are presented in Tables 7 and 8.

**Table 4:** Multiple Relationships Maintenance Scale items and CFA factor loadings from the full sample (*N* = 4,290)

		Loading
*Attraction disclosure*		
4	I talk with my partner(s) about my attractions to other people	.855
5	I tell my partner(s) about my intimate or flirtatious interactions with other people	.808
2	Throughout a relationship, I discuss with my partner(s) whether we may have sexual or romantic interactions with other people	.706
*Jealousy regulation*		
11	My partner(s) and I can talk openly with each other about jealousy	.857
12	My partner(s) and I do not communicate well about jealousy. (R)*	.763
10	My partner(s) and I share and discuss our experiences with jealousy	.679
3	I avoid talking to my partner(s) about the exclusiveness of our relationship (i.e., whether my partner and I may fall in love or have sex with other people). (R)	.529
*Childcare willingness*		
24	I would be willing to share my time and resources (e.g., money, housing space) with my partner(s’) children	.896
22	I would be willing to help my partner(s) care for their children	.883
23	I would be able to treat my partner(s’) children as if they were my own	.851
*Compersion*		
8	I would enjoy it if my partner(s) were romantically intimate with someone else	.908
7	I would enjoy it if my partner(s) were having sex with other people	.803
*Shared extra-pair sexuality*		
19	I would prefer to involve my current partner(s) in my sexual interactions with other people	.724
21	My sexual fantasies about other people include my partner(s)	.708
*Partner hierarchy*		
13	I tend to give some romantic or sexual partner(s) priority in my life	.708
14	I give some romantic or sexual partner(s) more influence over my life decisions	.695
15	I avoid treating some romantic or sexual partners as superior. (R)	.478
*Resource distribution*		
30	I do not often think about how much time I spend with my partner(s). (R)	.622
28	I think about how satisfied my partner(s) are with the amount of effort and attention I give them	.593
29	I consider how fairly I share material resources (e.g., money, food, shelter) with my partner(s)	.434
*Sexual health maintenance*		
16	I use safer sex tools (e.g., condoms, hormonal birth control) with my partner(s) to avoid unwanted sexual outcomes (e.g., unintended pregnancy, sexually transmitted infection)	.632
18	I would be willing to have unprotected sex with my partner(s) even if I were not completely sure that they were free of sexually transmitted infection or if an unwanted pregnancy might occur. (R)	.544
*Reputation management*		
26	I tend to hide my romantic or sexual involvement with my partner(s) from other people	.842
27	I openly talk about my relationships with other people (R)	.425

^*^(R) = reverse scored

**Table 5:** CFA model fit of the MRMS nine factor solution

Data	N	χ^2^(df)	RMSEA, 90% CI	CFI	TLI	SRMR
Total sample (all pooled)	4290^a^	1609.542 (194)	.041 (.039, .043)	.952	.938	.036
Subsample (male)	1243^b^	587.662 (196)	.040 (.036, .044)	.953	.940	.038
Subsample (female)	2221	951.341 (194)	.042 (.039, .045)	.951	.936	.038
Subsample (mono behavior)	2289	940.714 (194)	.041 (.038, .044)	.949	.934	.037
Subsample (multi behavior)	1668	697.361 (194)	.039 (.036, .043)	.957	.944	.035
Subsample (mono identity)	1596^c^	570.275 (195)	.035 (.031, .038)	.954	.940	.036
Subsample (multi identity)	1779^d^	753.004 (195)	.040 (.037, .043)	.950	.935	.036
Subsample (monogamous orientation)	1596	570.275 (195)	.035 (.031, .038)	.954	.940	.036
Subsample (polyamorous orientation)	1142	493.666 (194)	.037 (.033, .041)	.962	.951	.035
Subsample (open relationship orientation)	406^e^	344.546 (195)	.043 (.036, .051)	.942	.925	.044
Subsample (swinging orientation)	231^f^	281.564 (196)	.043 (.032, .054)	.920	.896	.060
Subsample (N. America)	830	484.900 (195)	.042 (.038, .047)	.950	.935	.041
Subsample (C/S. America)	575^ g^	456.511 (195)	.048 (.043, .054)	.926	.904	.054
Subsample (Europe)	2161	910.543 (194)	.041 (.039, .044)	.952	.938	.038
Subsample (US + Canada)	830^ h^	484.900 (195)	.042 (.038, .047)	.950	.935	.041
Subsample (Finland)	655^i^	525.428 (195)	.051 (.046, .056)	.937	.918	.050
Subsample (Poland)	116^j^	264.019 (194)	.056 (.037, .072)	.915	.890	.074
Subsample (Brazil)	568^ k^	453.863 (195)	.048 (.043, .054)	.925	.903	.055
Subsample (Italy)	1131	539.484 (194)	.040 (.036, .044)	.949	.934	.039
Subsample (English-speaking)	965^ l^	541.486 (195)	.043 (.039, .047)	.949	.934	.041
Subsample (English non-speaking)	2678	1103.150 (194)	.042 (.039, .044)	.948	.933	.037
Subsample (< 39)	679	402.450 (194)	.040 (.034, .045)	.954	.940	.042
Subsample (≥ 40)	3091	1198.101 (194)	.041 (.039, .043)	.953	.938	.037
Partner 1	2878	1589.523 (194)	.050 (.048, .052)	.941	.923	.046
Partner 2	1076	824.304 (194)	.055 (.051, .059)	.937	.917	.053

^a^The actual N of responses may differ for each variable, the responses for the total sample of N = 4290 were estimated with Full Information Maximum Likelihood method (thus missing data was estimated)

^b^The residual variance of Items 7 and 18 were fixed at zero in the male subsample due to negative, non-significant residual variance

^c^The residual variance of Item 18 was fixed at zero in the mono identity subsample due to negative, non-significant residual variance

^d^The residual variance of Item 7 was fixed at zero in the multi identity subsample due to negative, non-significant residual variance

^e^The residual variance of Items 18 was fixed at zero in the open relationship orientation subsample due to negative, non-significant residual variance

^f^The residual variance of Items 7 and 16 was fixed at zero in the swinging orientation subsample due to negative, non-significant residual variance

^g^The residual variance of Item 13 was fixed at zero in the Central and South American subsample due to negative, significant residual variance

^h^The residual variance of Item 7 was fixed at zero in the US and Canadian subsample due to negative, significant residual variance

^i^The residual variance of Item 16 was fixed at zero in the Finnish subsample due to negative, non-significant residual variance

^j^The residual variances of Items 11 and 18 were fixed at zero in the Polish subsample due to negative, non-significant and significant, respectively residual variances. The error variances of items 2 and 5 and items 10 and 12 (within their primary factors) had to be correlated to improve model fit

^k^The residual variance of Item 13 was fixed at zero due to negative, significant residual variance

^l^The residual variance of Item 7 was fixed at zero due to negative, significant residual variance

**Table 6:** Measurement invariance of the MRMS 9-factor model between gender, relationship behavior (mono vs multi), relationship identity (mono vs multi), relationship orientation (monogamous, polyamorous, open, swinging), continent (North-America, Central- and South-America, vs European), language (English- vs non-English speaking), and age (40 and below vs above 40)

Model	χ^2^ (df)	CFI	TLI	RMSEA (90% CI)	Δχ^2^ (Δdf)	ΔCFI	ΔTLI	ΔRMSEA
*Gender invariance*								
Configural	1543.767 (388)	.951	.936	.041 (.039–.044)				
Metric (loadings)	1577.072 (402)	.950	.937	.041 (.039–.043)	33.305 (14)	-0.001	− 0.001	< 0.001
Scalar (intercepts)	1634.131 (416)	.948	.937	.041 (.039– .043)	57.059 (14)	− 0.002	< 0.001	< 0.001
*Number of partners invariance*								
Configural	1626.366 (388)	.953	.939	.036 (.038, .042)				
Metric (loadings)	1718.005 (402)	.950	.937	.041 (.039, .043)	91.639 (14)	− 0.003	− 0.002	0.005
Scalar (intercepts)	2023.659 (416)	.939	.926	.044 (.042, .046)	305.654 (14)	− 0.011	− 0.011	0.003
Scalar partial (intercepts)^a^	1944.593 (417)	.942	.930	.043 (.041, .045)	226.588 (15)	− 0.008	− 0.007	0.002
*Relationship identity invariance*								
Configural	1329.440 (388)	.951	.937	.038 (.036, .040)				
Metric (loadings)	1430.279 (402)	.947	.933	.039 (.037, .041)	100.839 (14)	− 0.004	− 0.004	0.001
Scalar (intercepts)	1861.479 (416)	.925	.909	.045 (.043, .047)	431.200 (14)	− 0.022	− 0.024	0.006
Scalar partial (intercepts)^b^	1584.414 (414)	.940	.926	.041 (.039, .043)	154.135 (12)	− 0.007	− 0.007	− 0.004
*Continent of residence invariance*^*d*^								
Configural	1848.520 (585)	.948	.933	.043 (.040, .045)				
Metric (loadings)	1934.944 (613)	.946	.933	.043 (.040, .045)	86.424 (28)	− 0.002	< 0.001	< 0.001
Scalar (intercepts)	2403.832 (641)	.928	.914	.048 (.046, .050)	468.888 (28)	− 0.02	− 0.019	0.005
Scalar partial (intercepts)^e^	2164.379 (638)	.937	.925	.045 (.043, .047)	229.435 (25)	− 0.009	− 0.008	− 0.003
*Language invariance*^*f*^								
Configural	1629.835 (390)	.948	.933	.042 (.040, .044)				
Metric (loadings)	1664.554 (404)	.947	.934	.041 (.039, .043)	34.719 (14)	− 0.001	0.001	− 0.001
Scalar (intercepts)	1849.764 (418)	.940	.928	.043 (.041, .045)	185.210 (14)	− 0.007	− 0.006	0.002
*Age invariance*								
Configural	1625.746 (388)	.953	.938	.041 (.039, .043)				
Metric (loadings)	1641.856 (402)	.953	.940	.040 (.038, .042)	16.110 (14)	< 0.001	0.002	− 0.001
Scalar (intercepts)	1763.569 (416)	.948	.937	.041 (.039, .043)	121.713 (14)	− 0.005	− 0.003	0.001

^a^The residual variance of Items 7 was fixed at zero in the scalar model, and the intercept of Item 7 was freed across the mono and multi groups to achieve partial scalar invariance

^b^The intercepts of Items 2 and 13 were freed across the mono and multi groups

^c^The intercept of Item 2 was freed in the monogamous group, and the intercepts of Items 7, 11, and 13 were freed in the polyamorous group to achieve partial scalar invariance. The residual variance of Item 7 was fixed at zero in the scalar model

^d^The residual variance of Item 13 was fixed at zero in all models (i.e., configural, metric, and scalar)

^e^The intercepts of Items 2 and 30 were freed in the European group and the intercept of item 29 in the Central and South American group to achieve partial scalar invariance

^f^The residual variance of Item 7 was fixed at zero in all models

**Table 7:** Group means obtained from each multigroup comparison’s standardized scalar model, part 1

	Female	Multi behavior	Multi identity	English-speaking	Above 40
Attraction Disclosure	− 0.138***	1.010***	1.733***	1.045***	0.486***
Jealousy Regulation	0.190***	0.221***	0.535***	0.113**	− 0.004
Childcare Willingness	− 0.055	− 0.136***	− 0.187***	0.001	− 0.052
Compersion	− 0.423***	1.372***	1.860***	1.013***	0.594***
Shared Extra− pair Sexuality	− 0.669***	0.530***	1.118***	0.636***	0.455***
Partner Hierarchy	− 0.129**	− 0.080	0.404***	0.089	0.150
Resource Distribution	0.010	0.076	0.162***	0.403***	− 0.112
Sexual Health Maintenance	0.393***	0.252***	0.381***	0.002	− 0.157*
Reputation Management	− 0.444***	0.282***	0.308***	0.330***	0.299***

The group label pairs not presented here had a mean standardized to zero

* *p* < .05, ** *p* < .01, *** *p* < .001

**Table 8:** Group means obtained from each multigroup comparison’s standardized scalar model, part 2

	Polyamory	Open relationship	Swinging	C/S. America	Europe
Attraction disclosure	1.943***	1.117***	2.855***	− 0.971***	− 0.766***
Jealousy regulation	0.779***	0.207**	0.755***	0.082	− 0.185***
Childcare willingness	− 0.187***	− 0.309***	0.016	− 0.066	− 0.018
Compersion	2.733***	1.385***	2.890***	− 1.257***	− 0.954***
Shared Extra-pair sexuality	1.019***	1.133***	4.383***	− 0.919***	− 0.624***
Partner hierarchy	0.261***	0.498***	0.230*	0.350***	− 0.117**
Resource distribution	0.277***	− 0.187**	0.362**	− 0.425***	− 0.630***
Sexual health maintenance	0.550***	0.227**	0.125	0.258**	− 0.025
Reputation management	0.117*	0.603***	0.763***	− 0.389***	− 0.386***

The group label pairs not presented here had a mean standardized to zero. Polyamory, open relationship, and swinging were compared to monogamous relationships. Central and South America and Europe were compared to North America

^*^ *p* < .05. ** *p* < .01. *** *p* < .001

The nine factors, in order of variance explained, were: extra-pair attraction disclosure (*α* = .85; e.g., “I tell my partner about my intimate or flirtatious interactions with other people”), jealousy regulation (*α* = .71; e.g., “My partner(s) and I can talk openly with each other about jealousy”), childcare willingness (*α* = .90; e.g., “I would be willing to share my time and resources [e.g., money, housing space] with my partner(s’) children), compersion (*α* = .90; e.g., “I would enjoy it if my partner(s) were romantically/sexually intimate with someone else), shared extra-pair sexual experiences (*α* = .59; e.g., “My sexual fantasies about other people include my partner(s)”), partner hierarchy (*α* = .51; e.g., “I give some romantic or sexual partner(s) more influence over my life decisions”), thoughtfulness about resource distribution (*α* = .52; e.g., “I consider how fairly I share material resources [e.g., food, money, shelter] with my partner(s)”), sexual health maintenance (*α* = .48; e.g., I user safer sex tools [e.g., condoms, hormonal birth control] with my partner(s) to avoid unwanted sexual outcomes), and reputation management (*α* = .60; e.g., “I tend to hide my romantic or sexual involvement with my partner(s) from other people”). Items loaded on each factor as anticipated except for the items from the hypothesized factor “exclusivity agreement,” which unexpectedly loaded with “attraction disclosure,” and “jealousy regulation.” Mean ratings were computed for each component (see Table 9 for bivariate correlations among the MRMS factors).

**Table 9:** Bivariate correlations among MRMS-self factor scores

	Attraction disclosure	Jealousy regulation	Childcare willingness	Compersion	Shared sex		Resource hierarchy	Sexual distribution	Health
Attraction disclosure									
Jealousy regulation	.465**								
Childcare willingness	.038*	.113**							
Compersion	.660**	.219**	.014						
Shared sex	.522**	.264**	.109**	.469**					
Partner hierarchy	− .036*	− .023	.058*	− .077**	.074**				
Resource distribution	.196**	.242**	.243**	.099**	.186**	.123**			
Sexual health maint	.163**	.138**	− .050**	.106**	.001	− .110**	.032*		
Reputation management	− .096**	− .329**	− .111**	− .052*	− .061**	.029	− .151**	− .058**	

^*^p < .05 **p < .01

## Do People with More Consensual Non-Monogamy Experience Engage More Often in the MRMS Practices? *(RA4)*

To address *RA4*, we first collapsed polyamorous, swinging, and open relationship participants into a single “CNM” category. Then, we compared how scores on the MRMS differed across relationship identity (i.e., monogamous vs. CNM) using nine between-subject ANOVAs (Table 10). Our critical p-value was Bonferroni corrected to adjust for inflated error from multiple comparisons (*p* = .05/9 = .006). There were main effects for attraction disclosure, jealousy regulation, childcare willingness, compersion, shared extra-pair sexuality, partner hierarchy, sexual health maintenance, and reputation management. CNM people were more likely than monogamous people to report attraction disclosure, jealousy regulation, compersion, shared extra-pair sexuality, sexual health maintenance, and reputation management. People in monogamous relationships reported more partner hierarchy and more willingness to care for a partner’s children. There was also a marginal main effect for resource distribution, such that CNM participants report more thoughtfulness about resource distribution than monogamous participants, but this did meet our adjusted p-value.

**Table 10:** Means, standard deviations, and test statistics comparing monogamous and consensual non-monogamy participants’ scores on each MRMS-self factor score

	Monogamous		CNM		
	*M*	*SD*	*M*	*SD*	
Attraction Disclosure	2.75	1.60	5.46	1.48	*F*(1, 3301) = 2559.39, *p* < .001, *η*^*2*^ = .437
Jealousy Regulation	4.87	1.46	5.90	1.19	*F*(1, 3301) = 182.14, *p* < .001, *η*^*2*^ = .05
Childcare Willingness	5.10	1.75	4.76	1.91	F(1, 3301) = 28.13, p < .001, *η*^*2*^ = .01
Compersion	1.59	1.29	4.72	1.81	*F*(1, 3301) = 3216.01, *p* < .001, *η*^*2*^ = .493
Shared Sex	3.76	1.46	4.87	1.40	*F*(1, 3301) = 500.84, *p* < .001, *η*^*2*^ = .13
Partner Hierarchy	4.62	1.34	4.44	1.54	*F*(1, 3301) = 11.39, p = .001, *η*^*2*^ = .00
Resource Distribution	5.25	1.23	5.36	1.21	*F*(1, 3301) = 6.64, *p* = .010
Sexual Health Maint	4.97	1.84	5.48	1.65	*F*(1, 3301) = 67.90, *p* < .001, *η*^*2*^ = .02
Reputation Management	2.55	1.58	2.97	1.63	*F*(1, 3301) = 53.85, *p* < .001, *η*^*2*^ = .02

^*^p < .05 **p < .01

To further examine how the MRMS factor scores differed among people in CNM relationships, we performed nine between-subject ANOVAs comparing MRMS factor scores between people in polyamorous, open, and swinging relationships (Table 11). Our critical *p*-value was again Bonferroni corrected to adjust for inflated error from multiple comparisons (*p* = .05/9 = .006). There were main effects for attraction disclosure, jealousy regulation, childcare willingness, compersion, shared extra-pair sexuality, partner hierarchy, resource distribution, sexual health maintenance, and reputation management. Post hoc analyses with Bonferroni correction showed that swingers reported more attraction disclosure than people in polyamorous (*p* = .001) and open relationships (*p* < .001), and polyamorous reported more attraction disclosure than open (*p* < .001). Polyamorous (*p* < .001) and swinging (*p* < .001) reported more jealousy regulation than open. Swingers reported more willingness to care for their partner’s children than open (*p* = .001) and polyamorous (*p* = .022). People in open relationships reported less compersion than those in polyamorous (*p* < .001) and swinging (*p* < .001) relationships. Swingers reported more shared extra-pair sexuality than open (*p* < .001) and polyamorous (*p* < .001). Open reported more shared extra-pair sexuality than polyamorous (*p* = .002). People in polyamorous reported less partner hierarchy than those in open (*p* < .001) and swinging (*p* < .001). People in open relationships reported being less thoughtful about how resources are shared with partners compared to those in polyamorous (*p* < .001) and swinging (*p* < .001) relationships. Polyamorous reported more sexual health maintenance than open (*p* = .001) and swingers (*p* < .001). Finally, people in open (*p* < .001) and swinging (*p* < .001) relationships reported more reputation management than those in polyamorous relationships.

**Table 11:** Means, standard deviations, and test statistics comparing polyamorous, open, and swinging participants’ scores on each MRMS-self factor score

	Polyamorous		Open		Swinging		
	*M*	*SD*	*M*	*SD*	*M*	*SD*	
Attraction disclosure	5.55	1.38	4.92	1.73	5.93	1.14	*F*(2, 1753) = 42.54, *p* < .001, *η*^*2*^ = .05
Jealousy regulation	5.94	1.13	5.46	1.31	5.91	1.08	*F*(2, 1753) = 26.42, *p* < .001, *η*^*2*^ = .03
Childcare willingness	4.76	1.91	4.55	1.89	5.13	1.93	F(2, 1753) = 6.78, p = .001, *η*^*2*^ = .01
Compersion	4.99	1.75	3.94	1.86	4.75	1.60	F(2, 1753) = 53.10, p < .001, *η*^*2*^ = .01
Shared sex	4.62	1.31	4.81	1.44	6.23	0.92	*F*(2, 1753) = 144.76, *p* < .001, *η*^*2*^ = .14
Partner hierarchy	4.18	1.50	5.02	1.42	4.76	1.61	*F*(2, 1753) = 52.24, *p* < .001, *η*^*2*^ = .06
Resource distribution	5.46	1.15	5.02	1.31	5.51	1.18	F(2, 1753) = 22.28, p < .001, *η*^*2*^ = .03
Sexual health maint	5.62	1.56	5.27	1.73	5.15	1.86	F(2, 1753) = 11.77, p < .001, *η*^*2*^ = .01
Reputation management	2.73	1.55	3.35	1.66	3.45	1.76	*F*(2, 1753) = 33.43, *p* < .001, *η*^*2*^ = .04

^*^p < .05 **p < .01

Finally, to assess whether those with more CNM experience were more likely to engage in each MRMS practice, we performed bivariate correlations with a Holm-Bonferroni correction (Holland & Copenhaver, 1988) between participants’ MRMS factor scores and their self-reported experience practicing monogamy and each type of CNM (see Table 12). More experience with each type of CNM was associated with more attraction disclosure, jealousy regulation, compersion, shared extra-pair sexuality, and to a lesser extent thoughtfulness about how resources are shared with partners. More experience with monogamy was associated with less attraction disclosure, less compersion, and less shared extra-pair sexuality.

**Table 12:** Bivariate correlations among the MRMS-self factor scores and experience with each relationship type (*n* = 3761)

	Polyamory	Open relationships	Swinging	Monogamy
Attraction disclosure	.506*	.454*	.384*	− .159*
Jealousy regulation	.218*	.160*	.137*	− .019
Childcare willingness	− .032	− .034	.007	.005
Compersion	.579*	.475*	.376*	− .188*
Shared sex	.198*	.261*	.410*	− .063*
Partner hierarchy	− .120*	.028	.043	.044
Resource distribution	.079*	.041	.059*	.028
Sexual health maint	.087*	.035	− .016	− .031
Reputation management	− .023	.025	.071*	− .031

^*^p < .002

## Does Engagement in the MRMS Practices Relate to Relationship Quality? *(RA5)*

To address *RA5*, we used the relationship quality dataset (n = 871; see Table 1 for descriptives) to examine how engagement in each MRMS practice was related to each relationship quality outcome for partners 1 and 2.

We first performed bivariate correlations with a Holm-Bonferroni correction among the MRMS factors and each relationship quality outcome for partner 1 and partner 2 (see Table 13). To determine how each MRMS factor was related to relationship quality for people in CNM and monogamous relationships, we performed fifty-four moderated hierarchical regressions (27 for partner 1; 27 for partner 2) to compare how monogamous and CNM peoples’ nine MRMS factor scores related to three outcomes: 1) Overall relationship quality (i.e., total PRQC scores), 2) a composite measure of investment quality (i.e., scores on the investment scale, nurturance scale, and interdependence scale; partner 1: *α* = .60; partner 2: *α* = .80), and 3) a composite measure of sexual quality (i.e., scores on the NSSS, eroticism scale; partner 1: *α* = .80; partner 2: *α* = .73). Bonferroni corrections were used for each set of partner analyses (*p* = .05/27 = .002).

**Table 13:** Bivariate correlations among MRMS-partner factor scores and partner-specific relationship quality outcomes

	Attraction Disclosure	Jealousy Regulation	Childcare Willingness	Compersion	Shared Sex	Partner Hierarchy	Resource Distribution	Sexual Health	Reputation Management
*Partner 1 (n = 767)*									
PRQC Overall	.300*	.528*	.252*	.068	.377*	.323*	.325*	− .016	− .341*
Satisfaction	.332*	.509*	.171*	.126*	.302*	.178*	.240*	.009	− .287*
Commitment	.255*	.409*	.362*	.089	.253*	.370*	.401*	.086	− .381*
Intimacy	.252*	.476*	.217*	.047	.289*	.354*	.315*	− .004	− .338*
Trust	.206*	.386*	.204*	.109	.214*	.206*	.228*	.026	− .260*
Passion	.122	.298*	.032	− .094	.379*	.155*	.051	− .007	− .061
Love	.283*	.446*	.287*	.100	.299*	.323*	.405*	− .022	− .377*
Current Investment	.185*	.167*	.377*	.189*	.223*	.437*	.372*	− .229*	− .265*
Future Investment	.111	.168*	.086	.046	.185*	.137*	.129*	− .095	− .116
NSSS Self	.116	.288*	.115	− .044	.342*	.108	.080	− .012	− .060
NSSS Partner	.091	.297*	.024	− .094	.226*	.036	.011	.080	− .031
Eroticism	.067	.229	.000	− .119	.373*	.160*	.066	− .024	.002
Nurturance	.237*	.398*	.305*	.089	.231*	.368*	.387*	.002	− .342*
Interdependence Fac	.202*	.378*	.338*	.016	.243*	.337*	.328*	− .030	− .322*
Interdependence Inter	− .158*	− .276*	− .050	− .100	− .117	.068	− .066	.110	− .296*
*Partner 2 (n = 344)*									
PRQC Overall	.417*	.465*	.458*	.127	.372*	.418*	.614*	− .123	− .280*
Satisfaction	.408*	.419*	.149	.262*	.284*	.138	.242*	− .058	− .172
Commitment	.297*	.370*	.481*	.024	.254*	.482*	.650*	− .148	− .312*
Intimacy	.397*	.411*	.473*	.064	.285*	.450*	.609*	− .140	− .277*
Trust	.315*	.415*	.379*	.242*	.192*	.311*	.469*	− .044	− .258*
Passion	.179	.157	.102	− .024	.451*	.103	.170	− .104	.067
Love	.368*	.413*	.521*	.062	.266*	.433*	.685*	− .073	− .345*
Current Investment	.261*	.306*	.580*	.013	.101	.512*	.651*	− .089	− .360*
Future Investment	.194*	.250*	.466*	− .044	.191*	.420*	.529*	− .166	− .276*
NSSS Self	.129	.138	− .004	− .032	.305*	.126	.072	− .160	.020
NSSS Partner	.135	.236*	− .036	− .043	.284*	.099	.099	− .111	− .035
Eroticism	.111	.066	− .075	− .065	.428*	− .028	.003	− .122	.184
Nurturance	.366*	.436*	.509*	.092	.173	.431*	.668*	− .077	− .367*
Interdependence Fac	.308*	.418*	.470*	.068	.194*	.427*	.537*	− .158	− .239*
Interdependence Inter	− .203*	− .202*	.128	− .204*	− .062	.138	.096	− .066	.054

^*^p < .002

In Step 1 of each regression, relationship identity (dummy coded to monogamous and CNM) and an MRMS factor score were included as predictors (see Table 14 for test statistics). Standardized beta regression coefficients are reported below. For partner 1 and partner 2, overall relationship quality was predicted by attraction disclosure (*β*_*p1*_ = .42; *β*_*p2*_ = .38), jealousy regulation (*β*_*p1*_ = .54; *β*_*p2*_ = .47), childcare willingness (*β*_*p1*_ = .25; *β*_*p2*_ = .47), shared extra-pair sexuality (*β*_*p1*_ = .39; *β*_*p2*_ = .34), partner hierarchy (*β*_*p1*_ = .31; *β*_*p2*_ = .47), resource distribution (*β*_*p1*_ = .29; *β*_*p2*_ = .62), and reputation management (*β*_*p1*_ = -.35.; *β*_*p2*_ = -.28).

**Table 14:** Test statistics for each regression predicting relationship quality, investment quality, and sexual quality from each MRMS-partner factor score (Step 1) and the interaction with relationship identity (Step 2). Partner 2 interactions were not performed because there were insufficient monogamous participants for comparison. Critical *p* was set to *p* < .002 to adjust for multiple comparisons

	Outcome	Main effect	Interaction x relationship identity
*Partner 1*			
Attraction disclosure	Relationship quality	*R*^*2*^ = .12, *F*(2, 622) = 43.03, *p* < .001*	*R*^*2*^ = .13, *ΔF* (1, 621) = 5.87, *p* = .016
	Investment quality	*R*^*2*^ = .05, *F*(2, 637) = 17.01, *p* < .001*	*R*^*2*^ = .05, *ΔF* (1, 636) = 1.03, *p* = .311
	Sexual quality	*R*^*2*^ = .04, *F*(2, 632) = 13.05, *p* < .001*	*R*^*2*^ = .04, *ΔF* (1, 631) = 1.06, *p* = .304
Jealousy regulation	Relationship quality	*R*^*2*^ = .27, *F*(2, 622) = 115.13, *p* < .001*	*R*^*2*^ = .27, *ΔF* (1, 621) = 0.09, *p* = .762
	Investment quality	*R*^*2*^ = .08, *F*(2, 637) = 25.78, *p* < .001*	*R*^*2*^ = .08, *ΔF* (1, 636) = 0.55, *p* = .458
	Sexual quality	*R*^*2*^ = .10, *F*(2, 632) = 34.44, *p* < .001*	*R*^*2*^ = .10, *ΔF* (1, 631) = 0.08, *p* = .775
Childcare willingness	Relationship quality	*R*^*2*^ = .06, *F*(2, 622) = 20.75, *p* < .001*	*R*^*2*^ = .07, *ΔF* (1, 621) = 2.13, *p* = .145
	Investment quality	*R*^*2*^ = .13, *F*(2, 637) = 50.34, *p* < .001*	*R*^*2*^ = .16, *ΔF* (1, 636) = 14.37, *p* < .001*
	Sexual quality	*R*^*2*^ = .01, *F*(2, 632) = 3.75, *p* = .024	*R*^*2*^ = .11, *ΔF* (1, 631) = 0.74, *p* = .389
Compersion	Relationship quality	*R*^*2*^ = .01, *F*(2, 622) = 1.85, *p* = .158	*R*^*2*^ = .02, *ΔF* (1, 621) = 7.68, *p* = .006
	Investment quality	*R*^*2*^ = .03, *F*(2, 637) = 10.02, *p* < .001*	*R*^*2*^ = .03, *ΔF* (1, 636) = 0.04, *p* = .833
	Sexual quality	*R*^*2*^ = .01, *F*(2, 632) = 4.53, *p* = .011	*R*^*2*^ = .02, *ΔF* (1, 631) = 4.23, *p* = .040
Shared Sex	Relationship quality	*R*^*2*^ = .14, *F*(2, 622) = 52.34, *p* < .001*	*R*^*2*^ = .15, *ΔF* (1, 621) = 6.18, *p* = .013
	Investment quality	*R*^*2*^ = .08, *F*(2, 637) = 27.04, *p* < .001*	*R*^*2*^ = .08, *ΔF* (1, 636) = 1.65, *p* = .199
	Sexual quality	*R*^*2*^ = .17, *F*(2, 632) = 65.35, *p* < .001*	*R*^*2*^ = .19, *ΔF* (1, 631) = 14.37, *p* < .001*
Partner hierarchy	Relationship quality	*R*^*2*^ = .10, *F*(2, 622) = 32.51, *p* < .001*	*R*^*2*^ = .10, *ΔF* (1, 621) = 2.94, *p* = .087
	Investment quality	*R*^*2*^ = .24, *F*(2, 637) = 102.97, *p* < .001*	*R*^*2*^ = .24, *ΔF* (1, 636) = .01, *p* = .927
	Sexual quality	*R*^*2*^ = .02, *F*(2, 632) = 7.01, *p* = .001*	*R*^*2*^ = .02, *ΔF* (1, 631) = 0.70, *p* = .403
Resource distribution	Relationship quality	*R*^*2*^ = .08, *F*(2, 622) = 27.88, *p* < .001*	*R*^*2*^ = .09, *ΔF* (1, 621) = 2.34, *p* = .126
	Investment quality	*R*^*2*^ = .16, *F*(2, 637) = 59.09, *p* < .001*	*R*^*2*^ = .18, *ΔF* (1, 636) = 15.39, *p* < .001*
	Sexual quality	*R*^*2*^ = .01, *F*(2, 632) = 3.97, *p* = .019	*R*^*2*^ = .02, *ΔF* (1, 631) = 1.88, *p* = .171
Sexual Health Maint	Relationship quality	*R*^*2*^ < .01, *F*(2, 622) = 0.17, *p* = .845	*R*^*2*^ < .01, *ΔF* (1, 621) = 0.34, *p* = .562
	Investment quality	*R*^*2*^ = .03, *F*(2, 637) = 9.29, *p* < .001*	*R*^*2*^ = .03, *ΔF* (1, 636) = 1.20, *p* = .282
	Sexual quality	*R*^*2*^ = .01, *F*(2, 632) = 3.39, *p* = .034	*R*^*2*^ = .01, *ΔF* (1, 631) = 0.34, *p* = .559
Reputation Management	Relationship quality	*R*^*2*^ = .12, *F*(2, 622) = 40.64, *p* < .001*	*R*^*2*^ = .12, *ΔF* (1, 621) = 4.04, *p* = .045
	Investment quality	*R*^*2*^ = .10, *F*(2, 637) = 34.42, *p* < .001*	*R*^*2*^ = .10, *ΔF* (1, 636) = 0.21, *p* = .646
	Sexual quality	*R*^*2*^ = .01, *F*(2, 632) = 3.21, *p* = .041	*R*^*2*^ = .02, *ΔF* (1, 631) = 6.95, *p* = .009
*Partner 2*			
Attraction disclosure	Relationship quality	*R*^*2*^ = .14, *F*(2, 262) = 21.68, *p* < .001*	
	Investment quality	*R*^*2*^ = .09, *F*(2, 264) = 12.41, *p* < .001*	
	Sexual quality	*R*^*2*^ = .01, *F*(2, 261) = 1.81, *p* = .166	
Jealousy regulation	Relationship quality	*R*^*2*^ = .22, *F*(2, 262) = 36.23, *p* < .001*	
	Investment quality	*R*^*2*^ = .16, *F*(2, 264) = 25.96, *p* < .001*	
	Sexual quality	*R*^*2*^ = .01, *F*(2, 261) = 1.06, *p* = .349	
Childcare Willingness	Relationship quality	*R*^*2*^ = .22, *F*(2, 262) = 37.68, *p* < .001*	
	Investment quality	*R*^*2*^ = .35, *F*(2, 264) = 71.43, *p* < .001*	
	Sexual quality	*R*^*2*^ = .01, *F*(2, 261) = 0.99, *p* = .374	
Compersion	Relationship quality	*R*^*2*^ = .01, *F*(2, 262) = 1.17, *p* = .311	
	Investment quality	*R*^*2*^ = .01, *F*(2, 264) = 0.66, *p* = .519	
	Sexual quality	*R*^*2*^ = .01, *F*(2, 261) = 1.59, *p* = .205	
Shared sex	Relationship quality	*R*^*2*^ = .12, *F*(2, 262) = 17.13, *p* < .001*	
	Investment quality	*R*^*2*^ = .02, *F*(2, 264) = 3.28, *p* = .039	
	Sexual quality	*R*^*2*^ = .17, *F*(2, 261) = 26.49, *p* < .001*	
Partner hierarchy	Relationship quality	*R*^*2*^ = .21, *F*(2, 262) = 35.67, *p* < .001*	
	Investment quality	*R*^*2*^ = .29, *F*(2, 264) = 53.04, *p* < .001*	
	Sexual quality	*R*^*2*^ = .01, *F*(2, 261) = 0.85, *p* = .427	
Resource distribution	Relationship quality	*R*^*2*^ = .39, *F*(2, 262) = 83.05, *p* < .001*	
	Investment quality	*R*^*2*^ = .49, *F*(2, 264) = 126.79, *p* < .001*	
	Sexual quality	*R*^*2*^ = .01, *F*(2, 261) = 1.02, *p* = .360	
Sexual health maint	Relationship quality	*R*^*2*^ = .02, *F*(2, 262) = 3.24, *p* = .041	
	Investment quality	*R*^*2*^ = .02, *F*(2, 264) = 2.45, *p* = .089	
	Sexual quality	*R*^*2*^ = .02, *F*(2, 261) = 2.96, *p* = .054	
Reputation management	Relationship quality	*R*^*2*^ = .08, *F*(2, 262) = 10.81, *p* < .001*	
	Investment quality	*R*^*2*^ = .15, *F*(2, 264) = 22.63, *p* < .001*	
	Sexual quality	*R*^*2*^ = .04, *F*(2, 261) = 5.18, *p* = .006	

^*^p < .002

For partner 1 and partner 2, investment quality was predicted by attraction disclosure (*β*_*p1*_ = .27; *β*_*p2*_ = .29), jealousy regulation (*β*_*p1*_ = .28; *β*_*p2*_ = .40), childcare willingness (*β*_*p1*_ = .37; *β*_*p2*_ = .59), partner hierarchy (*β*_*p1*_ = .50; *β*_*p2*_ = .54), resource distribution (*β*_*p1*_ = .39; *β*_*p2*_ = .70), and reputation management (*β*_*p1*_ = -.31; *β*_*p2*_ = -.38). Partner 1 investment quality was further predicted by compersion (*β*_*p1*_ = .22), shared extra-pair sexuality (*β*_*p1*_ = .28) and sexual health maintenance (*β*_*p1*_ = -.16).

For partner 1 and partner 2, sexual quality was predicted by shared extra-pair sexuality (*β*_*p1*_ = .41; *β*_*p2*_ = .40). Partner 1 sexual quality was further predicted by attraction disclosure (*β*_*p1*_ = .21), jealousy regulation (*β*_*p1*_ = .31), partner hierarchy (*β*_*p1*_ = .11).

In Step 2, we included the interaction term (MRMS factor score × relationship identity). We could not test interactions for partner 2 because only twelve monogamous participants reported information about partner 2. Three partner 1 interactions were significant: willingness to provide childcare and investment quality, thoughtfulness about resource distribution and investment quality, and shared extra-pair sexual experiences and sexual quality. Simple slopes analyses were performed on these interactions using a bootstrapping method via PROCESS for SPSS (model 1; Hayes, 2017) to compare monogamous and CNM relationship quality across 16th (high), 50th (medium), and 84th (low) percentile MRMS factor scores. More childcare willingness was more strongly associated with higher investment quality in CNM relationships, *β*_*p1*_ = .22, *SE* = .02, *t*(638) = 10.36, *p* < .001, than in monogamous relationships, *β*_*p1*_ = .09, *SE* = .03, *t*(638) = 2.82, *p* = .005. More thoughtfulness about resource distribution was more strongly associated with higher investment quality in CNM relationships, *β*_*p1*_ = .42, *SE* = .04, *t*(638) = 10.97, *p* =  < .001, than in monogamous relationships, *β*_*p1*_ = .18, *SE* = .05, *t*(638) = 3.85, *p* < .001. More shared extra-pair sexual experiences was more strongly associated with higher sexual quality in CNM relationships, *β*_*p1*_ = .40, *SE* = .04, *t*(634) = 11.18, *p* < .001, than in monogamous relationships, *β*_*p1*_ = .18, *SE* = .05, *t*(634) = 3.97, *p* < .001.

## How Is Infidelity Related to Engagement in the MRMS Practices (*RA6*) and Relationship Quality (*RA7*)?

To address *RA6*, we averaged scores on the three infidelity questions for partner 1 and partner 2 to create composite infidelity measures (*α*_*p1*_ = .81; *α*_*p2*_ = .85). Bivariate correlations with a Holm-Bonferroni correction (see Table 15) showed that lower composite infidelity scores were related to higher scores on attraction disclosure, jealousy regulation, compersion, shared extra-pair sexuality, and sexual health, and lower scores on reputation management for both partner 1 and partner 2.

**Table 15:** Bivariate correlations between each MRMS-partner factor scores and how often their interactions with other people feel like infidelity in their relationship with each partner

		Infidelity partner 1	Infidelity partner 2
Attraction Disclosure	− .295**	− .384**	
Jealousy Regulation	− .470**	− .408**	
Childcare Willingness	− .006		− .015
Compersion	− .087		− .260**
Shared Sex	− .145**	− .161**	
Partner Hierarchy	.040		.074
Resource Distribution	− .040		− .013
Sexual Health Maint	− .126**	− .131**	
Reputation Management	.215**	.287**	

^*^p < .003

To address *RA7*, we performed bivariate correlations between the composite infidelity measures and the three composite relationship outcomes for each partner. Partner 1 infidelity was associated with lower relationship, *r*(765) = -.34, *p* < .001, and sexual quality, *r*(775) = -.20, *p* < .001, but not investment quality, *r*(758) = -.05, *p* = .154. Partner 2 infidelity was likewise associated with lower relationship, *r*(345) = -.18, *p* = .001, and sexual quality, *r*(343) = -.19, *p* < .001, but not investment quality, *r*(348) = .04, *p* = .467.

## Discussion

We identified nine practices for maintaining multiple intimate relationships. The MRMS showed adequate fit, and showed invariance across men and women, single- and multi-partnered people, those who identified as monogamous or CNM, English and non-English speakers, North Americans and Europeans, people younger and older than thirty-nine years, and first and second partners.

Six practices (i.e., disclosure of extra-pair attraction, communication about jealousy, compersion, shared extra-pair sexual experiences, sexual health maintenance, and reputation management) were more common in CNM compared to monogamous relationships, and two practices (i.e., childcare willingness and hierarchy) were more common in monogamous relationships compared to CNM relationships. Furthermore, people who reported more experience with CNM of any type reported more attraction disclosure, better ability to communicate jealousy to a partner, more enjoyment in a partner’s other intimate relationships (i.e., compersion), more shared extra-pair sexual experiences (e.g., sharing sexual fantasies; having shared sex with another person), and more thoughtfulness about partner resource distribution, whereas more experience with monogamy was related to less attraction disclosure, compersion, and shared extra-pair sexual experiences. This suggests that the MRMS quantifies individual differences in the relationship maintenance practices and experiences that can differ between monogamous and CNM relationships.

Scores on the MRMS were related to higher relationship quality. The practices related to higher quality across nearly all outcomes (e.g., satisfaction, commitment/interdependence, intimacy/nurturance/love) were jealousy regulation and shared extra-pair sexual experiences. By comparison, attraction disclosure, childcare willingness, hierarchy, and resource distribution were likewise related to more relationship investment, trust, nurturance, satisfaction, and positive experiences with interdependence, but not sexual satisfaction or passion. This suggests that the MRMS practices may primarily mitigate conflict among partners for time, attention, and resources by ensuring that each person is receiving from their partner(s) the investments that they need to find the relationship rewarding. In this way, the MRMS practices may buffer against attachment injury because partners explicitly discuss and anticipate how interactions with other people may threaten existing patterns of resource exchange, commitment, and wellbeing. This may explain why people who engaged in the MRMS practices also reported that their interactions with other people less often felt like infidelity. Infidelity occurs when someone withholds information about extra-pair interaction that a partner who would deem in violation to an established relationship agreement (see Mogilski et al., 2023). This is perceived as betrayal, and may cause the aggrieved partner to lose trust and feel less secure in the pair-bond. By prompting partners to communicate about and anticipate relationship threats, the MRMS practices may reduce how often interactions with other people feel duplicitous or unpredictable.

For example, disclosing attraction and then discussing jealousy may prompt partners to brainstorm and precaution against the risks of partner disinvestment or rivalry among partners. This may explain why people in CNM relationships think more often about their partner’s extra-pair interactions yet experience less emotionally reactive jealousy than people in monogamous relationships (Mogilski et al., 2019). Compersion may likewise indicate relationship quality insofar as it arises from learning (Hunter & Stockwell, 2022) that a metamour does not threaten the stability and satisfaction of a valued pair-bond (Flicker et al., 2022). However, compersion was only weakly connected to satisfaction with and current investment in partner 1, and satisfaction and trust with partner 2.

Sharing extra-pair sexual experiences with a partner (e.g., having a threesome; sharing sexual fantasies about other people) may similarly build trust that an established sexual relationship agreement is being upheld. Sharing these experiences may not only reduce uncertainty about the details of a partner’s extra-pair interactions, but could be sexually gratifying and validating for each partner. People in CNM relationships are more likely to report attraction to more than one gender (Haupert et al., 2017; Valentova et al., 2020), which may facilitate shared enjoyment of extra-pair sex. Mutual attraction to a metamour may also facilitate friendship or romance and thereby reduce rivalry.

Hierarchy may safeguard existing partner investments. More partner hierarchy was widely associated with better relationship quality in our sample, contrary to previous research showing lower quality in hierarchical relationships (e.g., Flicker et al., 2021), or no differences among people whose relationship agreements involved more vs. fewer restrictions on partner’s extra-pair interactions (Vilkin & Davila, 2023). Distinguishing primary bonds from other relationships may relieve competition among partners if biased resource allocation (e.g., giving one partner more control over your life decisions) is mutually acknowledged and deemed acceptable to all partners. However, whether this is satisfying may depend on whether it complements each partner’s relationship preferences. For example, if a partner is considered “secondary”, and they desire more independence or less commitment, they may be more satisfied to adopt hierarchy. People in CNM relationships are more likely to report hierarchy (Balzarini et al., 2019a, 2019b, 2019c), so this may be a helpful practice for most people. On the other hand, if a partner wishes to be treated as an equally invested relationship, they may be less satisfied with hierarchy. Future research should address how partners perceive hierarchy within their relationship(s) and when hierarchical vs. egalitarian structures facilitate better relationship quality.

Greater willingness to care for a partner’s children may signal a partner’s continued or eventual involvement in childcare. However, it should be noted that we did not record actual investment in childcare. Some people in CNM relationships report non-normative family structures such as a polycule living in a shared house (Alarie, 2024; Wauthier, 2024), though it is unclear how often CNM families deviate from nuclear-family arrangements (i.e. two parents living with their children). For example, relationships that practice more hierarchy may more likely resemble monogamous families in the degree to which partners invest in children. Future research could clarify childcare provisioning agreements (e.g., whether extra-pair partners are expected to help or participate in the child’s life) and whether some agreements produce higher quality relationships or childcare outcomes.

Sexual health maintenance was only associated with current investment in partner 1, such that people who reported less concern for sexual health risk also reported less investment. Prior research shows mixed associations between relationship quality and contraceptive use (e.g., Cox et al., 2013; Williams et al., 2012; Wilson & Koo, 2008). Perhaps people who are less invested in their current partner are less vigilant about protecting their partner from the consequences of STIs or unplanned pregnancy. However, rather than protect or improve relationship satisfaction per se, this practice may be more consequential to partners’ physical safety and health.

Finally, people who reported more reputation management reported worse relationship quality. Being secretive about relationship(s) was related to less commitment, intimacy, trust, love, investment, nurturance, and fewer positive experiences with interdependence. Interestingly, it was also related to fewer negative experiences with interdependence, too. Possibly, this explains why people choose secretive relationships despite lower quality: being secretive allows a person to limit how much an extra-pair partner interferes in their life.

Somewhat surprisingly, most MRMS practices were related to relationship quality in both monogamous and CNM relationships. One possibility is that people in monogamous relationships benefit from these practices if they generally improve communication about relationship threats, or if they allow partners to avoid infidelity. Infidelity prevention is typically achieved through behaviors which dissuade a partner from extra-pair interactions (i.e., mate retention; Starratt, 2023), such as partner sequestration, public possessiveness, partner or rival derogation, gift-giving, and appearance enhancement. The MRMS practices may provide an alternative approach to resolving the threat of partner infidelity: allow certain kinds of extra-pair interactions but manage the risk introduced by these other relationship(s). Within monogamous relationships, communicating with a partner about extra-pair attraction could prompt a conversation about jealousy, even if partners then agree to maintain sexual exclusivity. Sharing information about extra-pair attractions or desires may reduce uncertainty about how these interactions will undermine relationship quality. Also, perhaps some of our ‘monogamous’ participants had more than one partner but yet considered themselves ‘monogamous’ by mainstream cultural standards (e.g., when couples “only play together” with others, or have an occasional threesome), in which case the MRMS practices could be relevant to someone who identifies as monogamous.

Three practices were more strongly associated with relationship quality in CNM compared to monogamous relationships. For partner 1, willingness to care for children and thoughtfulness about resource distribution was a stronger predictor of higher investment quality in CNM than monogamous relationships. Monogamous relationship agreements often implicitly assume that partners will preferentially allocate resources and childcare to each other, but having multiple relationships makes this less clear. Therefore, those who explicitly discuss and negotiate childcare and resource sharing may be more satisfied with investment quality if doing so clarifies each partner’s expectations for how resources will be shared. Likewise, shared extra-pair sexual experiences were more strongly related to higher sexual quality in CNM than in monogamous relationships. Similarly, people with multiple partners may benefit more from discussing how sexual interactions happen with other people if doing so reduces uncertainties about how sex occurs among partner, or if partners mutually enjoy the experience.

## Limitations and Broader Impacts

The MRMS achieved adequate model fit, but several of the nine practices showed low inter-item reliability (i.e., shared extra-pair sexuality, partner hierarchy, thoughtful resource distribution, sexual health maintenance, and reputation management). The current items that comprise these factors may not capture enough variability in the intended behavior, or they may not cohesively represent the same relationship maintenance practice. For example, shared extra-pair sexuality includes items such as “I tell my sexual fantasies to my partner” but also “my sexual fantasies about other people include my partner(s).” Though sharing sexual fantasies can facilitate a partner’s involvement, they may not actually become involved. For this reason, the MRMS should be most valued as a list of common, seemingly effective CNM relationship maintenance practices with questions for measuring engagement in these practices. But researchers should consider altering the items to suit their research questions or to improve their psychometric utility.

Furthermore, we showed that these nine practices are correlated with relationship quality, but our data do not demonstrate causality. For example, maybe more attraction disclosure, compersion, and shared sex lead to better relationships–but maybe people in better relationships are more open to attraction disclosure, compersion, and shared sex. Follow-up research should employ a placebo-controlled experimental design to evaluate how adopting the MRMS practices impacts relationship quality relative to other interventions. Likewise, a longitudinal design could help show that adopting these practices has an enduring causal influence on relationship quality.

Basic research on intimate relationships should consider how people with multiple relationships navigate the challenges of having several concurrent intimate partners. Social monogamy is the predominant norm from which relationship scientists reason about conflict resolution in relationships. The MRMS offers a succinct 30-item scale to reveal how people who consent to having multiple relationships navigate multi-partnering. However, before the MRMS practices can be recommended therapeutically, researchers must examine its associations with other established measures of personality traits (e.g., the Big Five or HEXACO), cognitive traits (e.g. general intelligence, social-cognitive abilities), and relationship quality. Further validating the MRMS could assist clinical researchers, therapists, and public health researchers who attempt to intervene with individuals and relationships struggling with non-monogamy. Similarly, marriage and family policy might develop MRMS-informed interventions to address public health issues associated with real or suspected multi-partnering within monogamous relationships (e.g., family dissolution, intimate partner violence, spread of STIs), or to educate people about common misconceptions of CNM (see Moors et al., 2021).

### Conclusion

Over the last few decades, people in CNM relationships have developed many strategies to manage the challenges of multi-partnering – but some of these might work better than others. We identified nine practices by which people in CNM relationships try to avoid or resolve potential conflicts caused by multi-partnering. Rather than ignore, sabotage, or keep secret a partner’s extra-pair relationships, people who practice CNM disclose these interactions to their partner(s) and work with their partner(s) to prevent or address the challenges that multiple relationships introduce. This paper is the first to develop and validate a standard, comprehensive list of multiple relationship maintenance practices, and to examine their associations with measures of relationship quality. As CNM becomes more common worldwide, it will be important for relationship researchers and clinicians to validate and offer such evidence-based safety guidelines for practicing non-monogamy.

## Data Availability

The study’s dataset may be accessed on the Open Science Framework (https://osf.io/wex2f).

## Multiple Relationships Maintenance Scale—Self (MRMS-Self)

Below is a series of statements. Please rate how well each statement generally describes you.

(1 = Does not describe me at all; 7 = Describes me extremely well).

1. Near the start of a relationship, I speak with my partner(s) about whether we may have sexual or romantic interactions with other people.
2. Throughout a relationship, I discuss with my partner(s) whether we may have sexual or romantic interactions with other people.
3. I avoid talking to my partner(s) about the exclusiveness of our relationship (i.e., whether my partner and I may fall in love or have sex with other people). (reverse-coded)
4. I talk with my partner(s) about my attractions to other people.
5. I tell my partner(s) about my intimate or flirtatious interactions with other people.
6. When I have an emotionally or sexually intimate experience with someone else, I tend to minimize or hide the experience from my partner(s). (reverse-coded)
7. I would enjoy it if my partner(s) were having sex with other people.
8. I would enjoy it if my partner(s) were romantically intimate with someone else.
9. The thought of my partner having sex with someone else arouses me.
10. My partner(s) and I share and discuss our experiences with jealousy.
11. My partner(s) and I can talk openly with each other about jealousy.
12. My partner(s) and I do not communicate well about jealousy. (reverse-coded)
13. I tend to give some romantic or sexual partner(s) priority in my life.
14. I give some romantic or sexual partner(s) more influence over my life decisions.
15. I avoid treating some romantic or sexual partners as superior. (reverse-coded)
16. I use safer sex tools (e.g., condoms, hormonal birth control) with my partner(s) to avoid unwanted sexual outcomes (e.g., unintended pregnancy, sexually transmitted infection).
17. I would get tested if there were a chance that I might contract a sexually transmitted infection from my partner(s).
18. I would be willing to have unprotected sex with my partner(s) even if I were not completely sure that they were free of sexually transmitted infection or if an unwanted pregnancy might occur. [reverse-coded]
19. I would prefer to involve my current partner(s) in my sexual interactions with other people.
20. I tell my sexual fantasies to my partner(s).
21. My sexual fantasies about other people include my partner(s).
22. I would be willing to help my partner(s) care for their children.
23. I would be able to treat my partner(s’) children as if they were my own.
24. I would be willing to share my time and resources (e.g., money, housing space) with my partner(s’) children.
25. I tend not to talk about my current partner(s) around people that I find attractive.
26. I tend to hide my romantic or sexual involvement with my partner(s) from other people.
27. I openly talk about my relationships with other people. (reverse-coded)
28. I think about how satisfied my partner(s) are with the amount of effort and attention I give them.
29. I consider how fairly I share material resources (e.g., money, food, shelter) with my partner(s).
30. I often think about how much time I spend with my partner(s).

## Multiple Relationships Maintenance Scale—Partner (MRMS-Partner)

Below is a series of statements. Please rate how well each statement generally describes your relationship with [partner name].

(1 = Does not describe our relationship at all; 7 = Describes our relationship extremely well).

1. Near the start of our relationship, I spoke with X about whether we may have sexual or romantic interactions with other people.
2. Throughout our relationship, I’ve discussed with X whether we may have sexual or romantic interactions with other people.
3. I avoid talking to X about the exclusiveness of our relationship (i.e., whether my partner and I may fall in love or have sex with other people). (reverse-coded)
4. I talk with X about my attractions to other people.
5. I tell X about my intimate or flirtatious interactions with other people.
6. When I have an emotionally or sexually intimate experience with someone else, I tend to minimize or hide the experience from X. (reverse-coded)
7. I would enjoy it if X were having sex with other people.
8. I would enjoy it if X were romantically intimate with someone else.
9. The thought of X having sex with someone else arouses me.
10. X and I share and discuss our experiences with jealousy.
11. X and I can talk openly with each other about jealousy.
12. X and I do not communicate well about jealousy. (reverse-coded)
13. I tend to give X romantic or sexual priority in my life.
14. I give X more influence over my life decisions.
15. I avoid treating X as romantically or sexually superior to other people. (reverse-coded)
16. I use safer sex tools (e.g., condoms, hormonal birth control) with X to avoid unwanted sexual outcomes (e.g., unintended pregnancy, sexually transmitted infection).
17. I would get tested if there were a chance that I might contract a sexually transmitted infection from X.
18. I would be willing to have unprotected sex with X even if I weren’t completely sure that they were free of sexually transmitted infection or if an unwanted pregnancy might occur. [reverse-coded]
19. I would prefer to involve X in my sexual interactions with other people.
20. I tell my sexual fantasies to X.
21. My sexual fantasies about other people include X.
22. I would be willing to help X care for their children.
23. I would be able to treat X’s children as if they were my own.
24. I would be willing to share my time and resources (e.g., money, housing space) with X’s children.
25. I tend not to talk about X around people that I find attractive.
26. I tend to hide my romantic or sexual involvement with X from other people.
27. I openly talk about my relationship with X with other people. (reverse-coded)
28. I think about how satisfied X is with the amount of effort and attention I give them.
29. I consider how fairly I share material resources (e.g., money, food, shelter) with X.
30. I often think about how much time I spend with X.
